# A Rare Case of Endotracheal Metastases in Head and Neck Squamous Cell Carcinoma: A Case Report and Literature Review

**DOI:** 10.7759/cureus.76903

**Published:** 2025-01-04

**Authors:** Sathish Krishnan, Faheem Abbasi, Praveen Jayapal, Dhileepan Selvarajan

**Affiliations:** 1 Pulmonary and Critical Care Medicine, Community Health Network, Indianapolis, USA; 2 Radiology, Lucile Packard Children's Hospital, Stanford University, Palo Alto, USA; 3 Critical Care Medicine, Thiruvarur Medical Center, Thiruvarur, IND

**Keywords:** chronic cough, endobronchial metastasis, endotracheal, endotracheal metastasis, flexible bronchoscopy, head and neck neoplasms, hoarse voice, subglottic cancer, tracheal tumour, vocal cord cancer

## Abstract

Head and neck squamous cell carcinoma (HNSCC) typically originates from the squamous cells lining the mucosal surfaces of the head and neck. Patients may present with diverse symptoms, including hoarseness of voice, difficulty swallowing (dysphagia), a neck mass, or a cough. While metastasis is usually regional, distant metastases, including tracheobronchial involvement, though rare, can occur and are often associated with a poor prognosis.

Here, we report the case of a 64-year-old patient with a history of smoking who presented with complaints of exertional dyspnea and a chronic cough for six months. Pulmonary function tests confirmed a diagnosis of chronic obstructive pulmonary disease (COPD), and bronchodilator therapy with ipratropium was initiated. Four months later, at a follow-up, the patient reported worsening cough and new-onset hoarseness. A CT scan of the neck revealed a lesion on the left vocal cord, and a flexible nasopharyngolaryngoscopy confirmed a left vocal cord tumor. A biopsy and elective tracheostomy were performed, with pathology demonstrating an invasive, moderately differentiated squamous cell carcinoma. A positron emission tomography-computed tomography (PET-CT) scan showed intense fluorodeoxyglucose (FDG) uptake in the vocal cord lesion and bilateral cervical lymph nodes, leading to a diagnosis of stage IV laryngeal cancer (T3N2cM0).

The patient underwent concurrent chemotherapy with cisplatin for seven weeks and radiation therapy targeted at the larynx and bilateral neck lymph nodes. A follow-up laryngoscopy and CT scan of the neck, five months post-diagnosis, showed near-complete resolution of the left vocal cord tumor and a reduction in the size of the cervical lymph nodes. Another PET-CT scan, performed six months post-diagnosis, showed no FDG uptake in the left vocal cord and cervical lymph nodes. However, a small focus of FDG uptake was noted in the upper posterolateral aspect of the tracheoesophageal stripe, which was reported as a tracheoesophageal lymph node. An esophagogastroduodenoscopy (EGD) with endoscopic ultrasound (EUS) was planned. However, after a thorough review of PET-CT scan images and discussion at the multidisciplinary team (MDT) meeting, the patient underwent a bronchoscopy instead. This revealed two small endotracheal lesions, confirmed by biopsy as invasive, moderately differentiated keratinizing squamous cell carcinoma.

Despite an excellent local response, the patient developed endotracheal metastasis, an uncommon occurrence. This case underscores the complexities in diagnosing head and neck squamous cell carcinoma (HNSCC) with atypical metastatic presentations. It highlights the necessity of an integrated approach for timely diagnosis and expeditious treatment.

## Introduction

The squamous cells lining the tissues of the head and neck region, including the oral cavity, pharynx, and larynx, are the origin of head and neck squamous cell carcinoma (HNSCC) [1]. Globally, it is the seventh most common cancer, with approximately 890,000 new cases and 450,000 deaths annually [2].

HNSCC emerges from a complex interplay of risk factors, including tobacco use, alcohol consumption, human papillomavirus (HPV), Epstein-Barr virus (EBV), and areca nut use, each contributing to varying extents based on geographic and demographic factors [3]. Additional influences encompass occupational exposures, poor oral hygiene, immunodeficiency states, and genetic predispositions like Fanconi anemia. Protective factors, such as diets rich in fruits and vegetables counterbalance risks like preserved meats and prior radiation exposure, highlighting the multifactorial nature of HNSCC etiology [1,4,5].

The development of distant metastases significantly worsens prognosis. The lungs, bones, and liver are the common metastatic sites of HNSCC while tracheobronchial metastases are rare and can occur through hematogenous, lymphatic, or direct spread [6]. This can present significant diagnostic challenges.

Treatment of HNSCC with tracheobronchial metastases typically involves systemic therapies, such as chemotherapy and immunotherapy, complemented by local treatments like radiotherapy or surgery. Immunotherapy, particularly for PD-L1-positive tumors, has shown promising outcomes [7]. However, the prognosis remains poor, with median survival often under 12 months, depending on the extent of metastasis, patients’ performance status, and response to treatment [8].

We report the case of a 64-year-old patient with a history of chronic smoking and COPD who developed a malignant tumor of the left vocal cord. The patient was treated with concurrent chemoradiotherapy and demonstrated a favorable local response. However, the disease progressed with the emergence of endotracheal metastases.

## Case presentation

**Figure 1:**
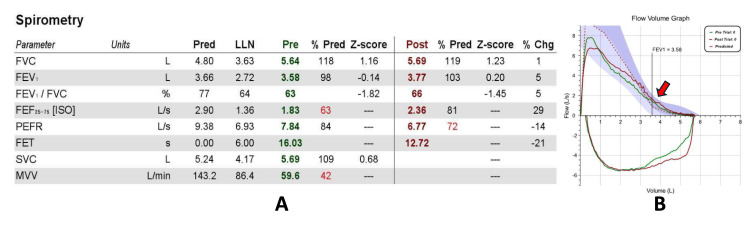
A: Spirometry measurements show a reduced FEV1/FVC ratio with no improvement with bronchodilator administration. B: Flow volume loops demonstrating a ‘coved’ pattern of expiratory limb (red arrow), seen in peripheral or lower airway obstruction. FEV1: Forced Expiratory Volume in 1 sec; FVC: Forced Vital Capacity

A 64-year-old male with 45 pack-years smoking history presented for the management of newly diagnosed chronic obstructive pulmonary disease (COPD). The patient experienced progressive exertional dyspnea and cough over the past six months. A spirometry and flow volume loop indicated a mild fixed obstructive ventilatory defect (Figure 1). He was started on inhaled tiotropium and as-needed albuterol inhalers.

**Figure 2:**
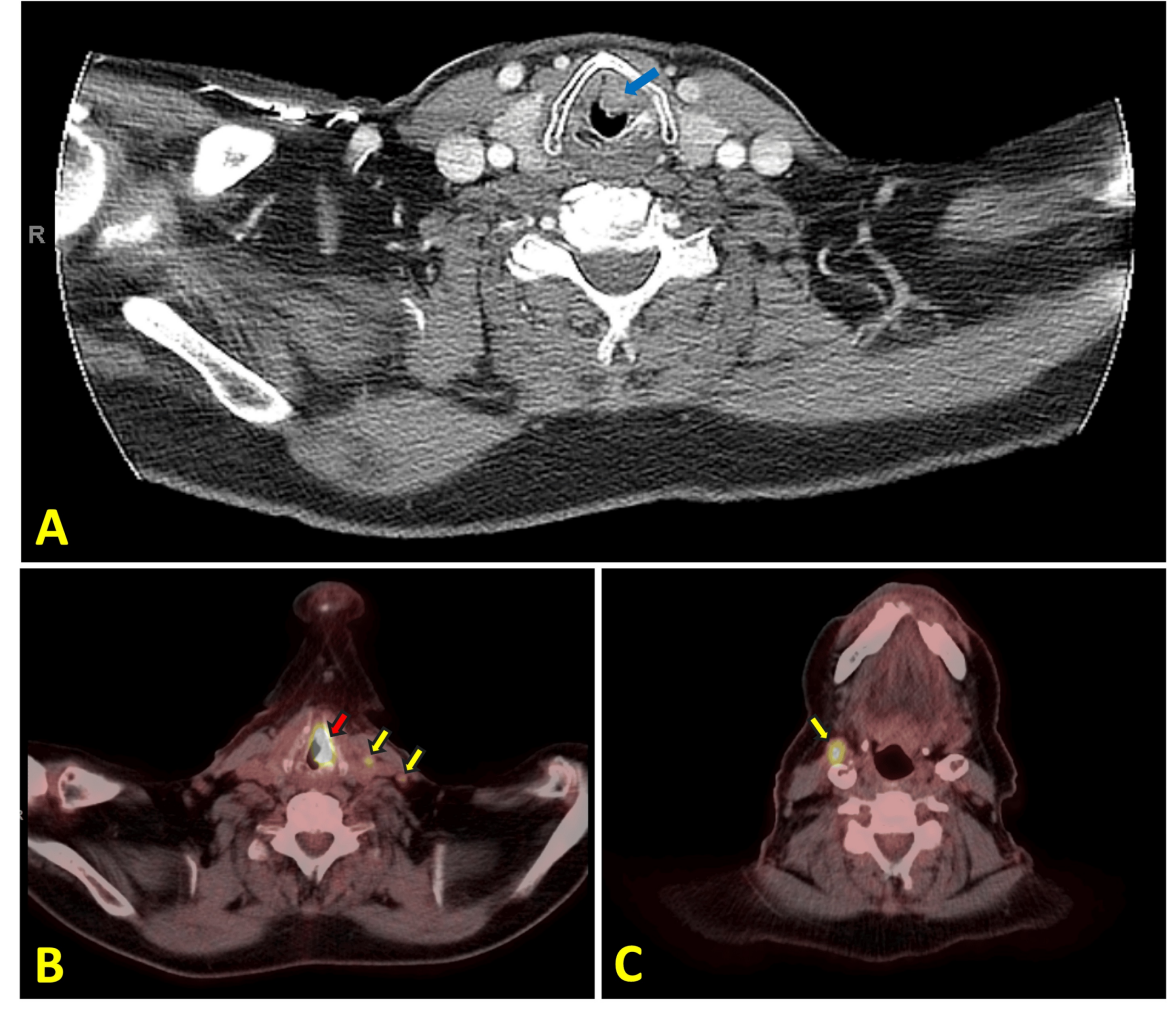
A: CT scan of the neck with a blue arrow indicating the polypoid lesion of the left vocal cord. B: PET-CT scan of laryngeal cancer with a red arrow showing FDG uptake at the left vocal cord and yellow arrows showing two normal-sized FDG-avid lymph nodes. C: PET-CT scan of neck with a yellow arrow showing a right upper anterior cervical FDG-avid lymph node, measuring 1.3 cm PET-CT: Positron Emission Tomography-Computed Tomography; FDG: Fludeoxyglucose

**Figure 3:**
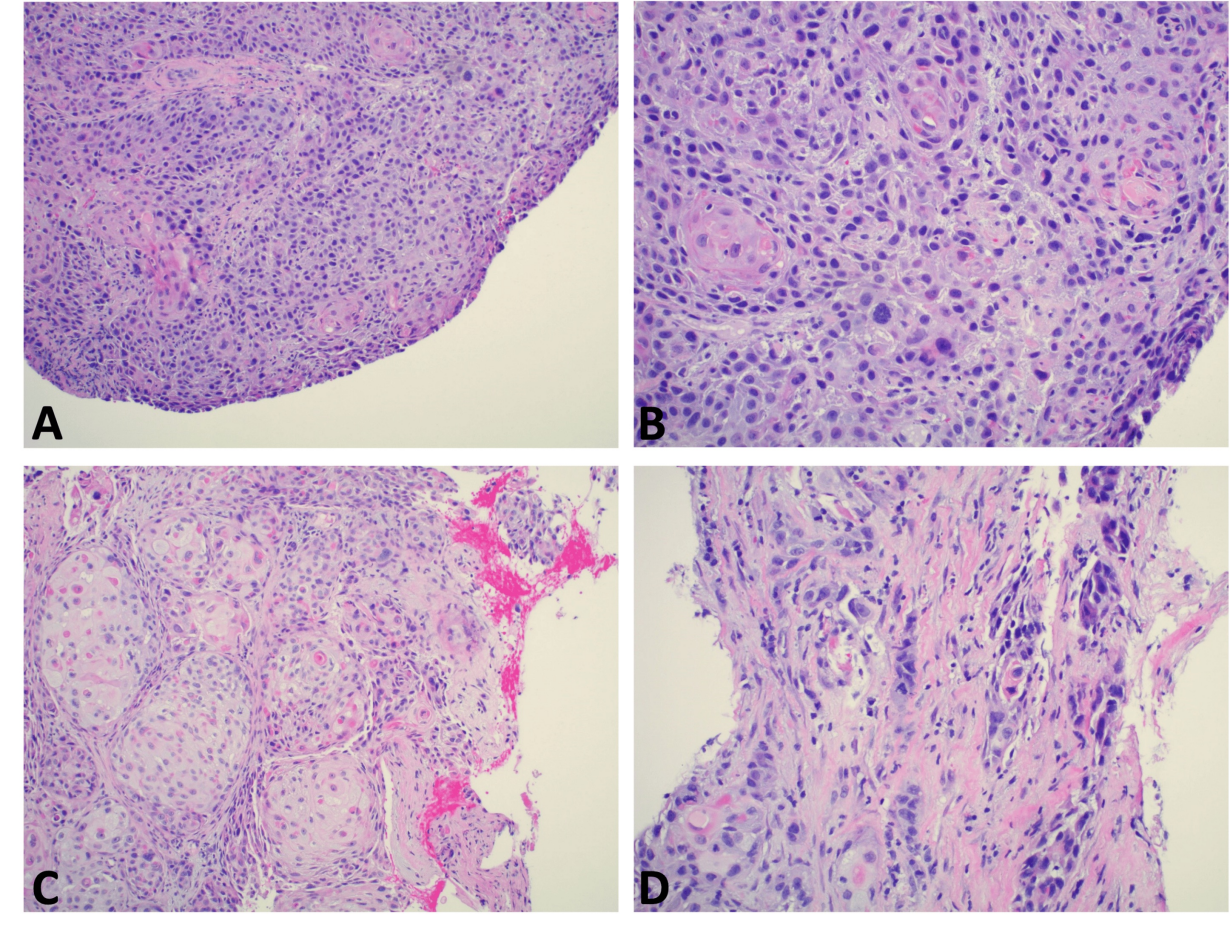
Upper Panel: Biopsy from vocal cord tumor. A: Low-power image shows proliferation of atypical squamous cells and keratin pearl formation. B: High-power image demonstrates keratin pearl formation, intercellular bridges, and pleomorphic nuclei, characteristic of invasive keratinizing squamous cell carcinoma. Lower Panel: Biopsy from the endotracheal lesion: C: Low-power image shows nests of malignant squamous cells with keratin pearl formation. D: High-power image demonstrates infiltrating groups of tumor cells, surrounded by desmoplastic stroma, characteristic of invasive keratinizing squamous cell carcinoma.

During a follow-up appointment four months later, the patient reported new-onset hoarseness and a worsening cough, despite improvement in dyspnea. Given that tiotropium can potentially cause throat-related side effects, such as hoarseness, sore throat, and dry throat, his symptoms were initially attributed to the bronchodilator. Consequently, ipratropium was temporarily discontinued for two weeks. However, the patient's symptoms did not improve during this period, indicating that the hoarseness and cough were likely unrelated to the medication and warranting further workup. Diagnostic evaluation began with a CT (computed tomography) scan of the neck, which revealed a small polypoid lesion originating from the left vocal fold (Figure 2). Flexible nasopharyngolaryngoscopy confirmed the presence of a tumor on the left vocal cord. The patient then underwent a direct laryngoscopy with a biopsy of the tumor and an elective tracheostomy. Biopsy results confirmed invasive moderately differentiated keratinizing squamous cell carcinoma (Figure 3). Additional imaging, including a CT scan of the neck and PET-CT (positron emission tomography-computed tomography), established stage IV subglottic cancer, classified as cT3N2cM0 (Figure 2).

The patient underwent radiation therapy to the larynx and bilateral neck lymph nodes, receiving a total radiation dose of 70 gray administered in 35 fractions, with cisplatin given weekly for 7 weeks. Five months post the initial diagnosis, a follow-up flexible nasopharyngolaryngoscopy was performed, revealing no focal lesion. A CT scan of the neck, performed at the same interval, showed a reduction in the size of the primary tumor involving the glottis, as well as a decrease in the size of the cervical lymph nodes (Figure 3). The patient's recovery progressed well, allowing for successful decannulation from the tracheostomy tube. A three-week follow-up examination after decannulation showed a well-healed tracheostomy stoma with no stridorous breath sounds. Importantly, the patient was able to resume oral feeding without experiencing dysphagia.

**Figure 4:**
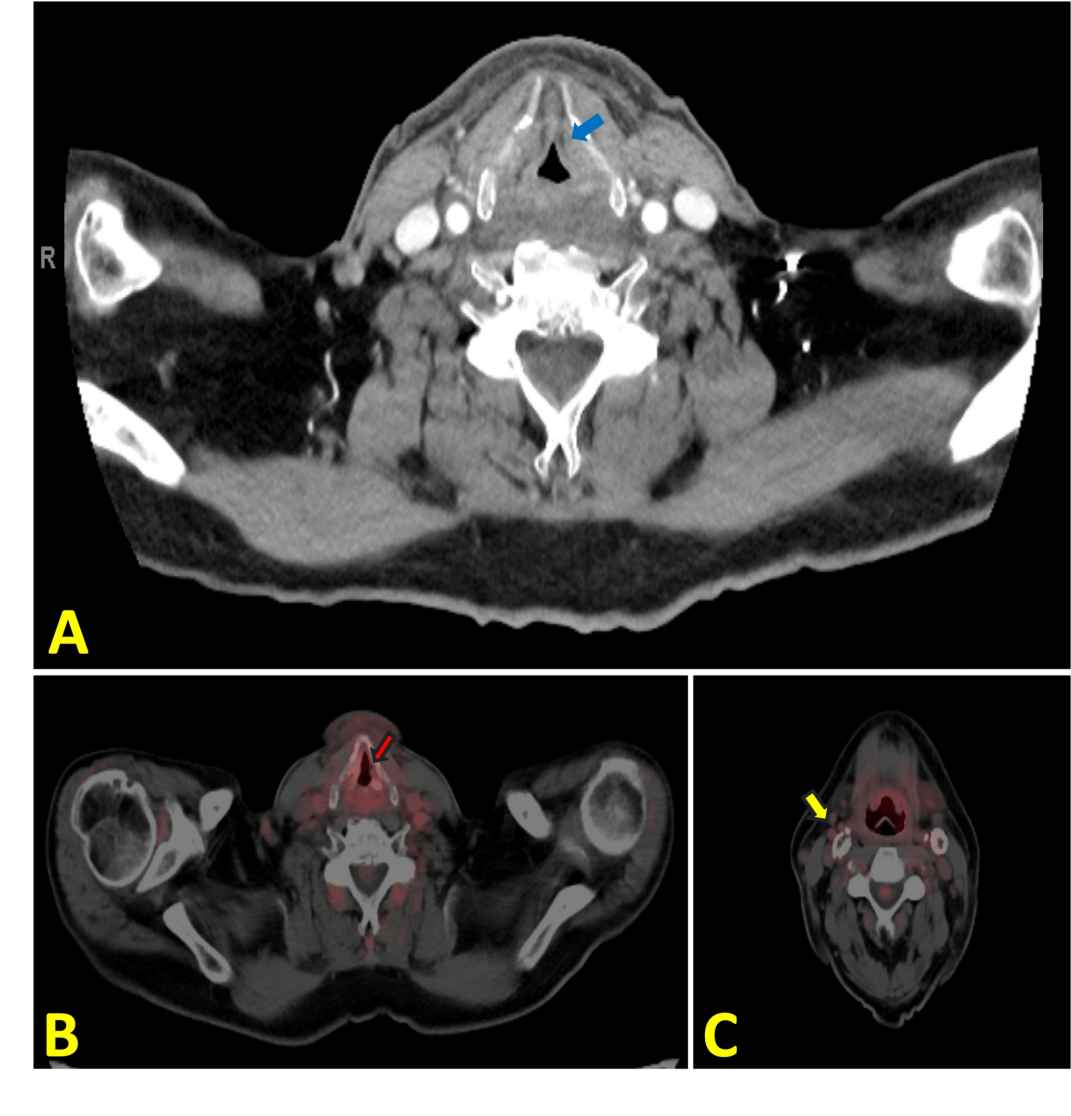
A: Post-treatment CT scan of laryngeal cancer with a blue arrow indicating a decrease in size of the left vocal cord lesion. B: Post-treatment PET-CT scan with a red arrow indicating improvement and resolution of FDG uptake in the laryngeal region. C: Post-treatment PET-CT scan with a yellow arrow indicating a decrease in size and resolution of FDG uptake in the right cervical node PET-CT: Positron Emission Tomography-Computed Tomography; FDG: Fludeoxyglucose

**Figure 5:**
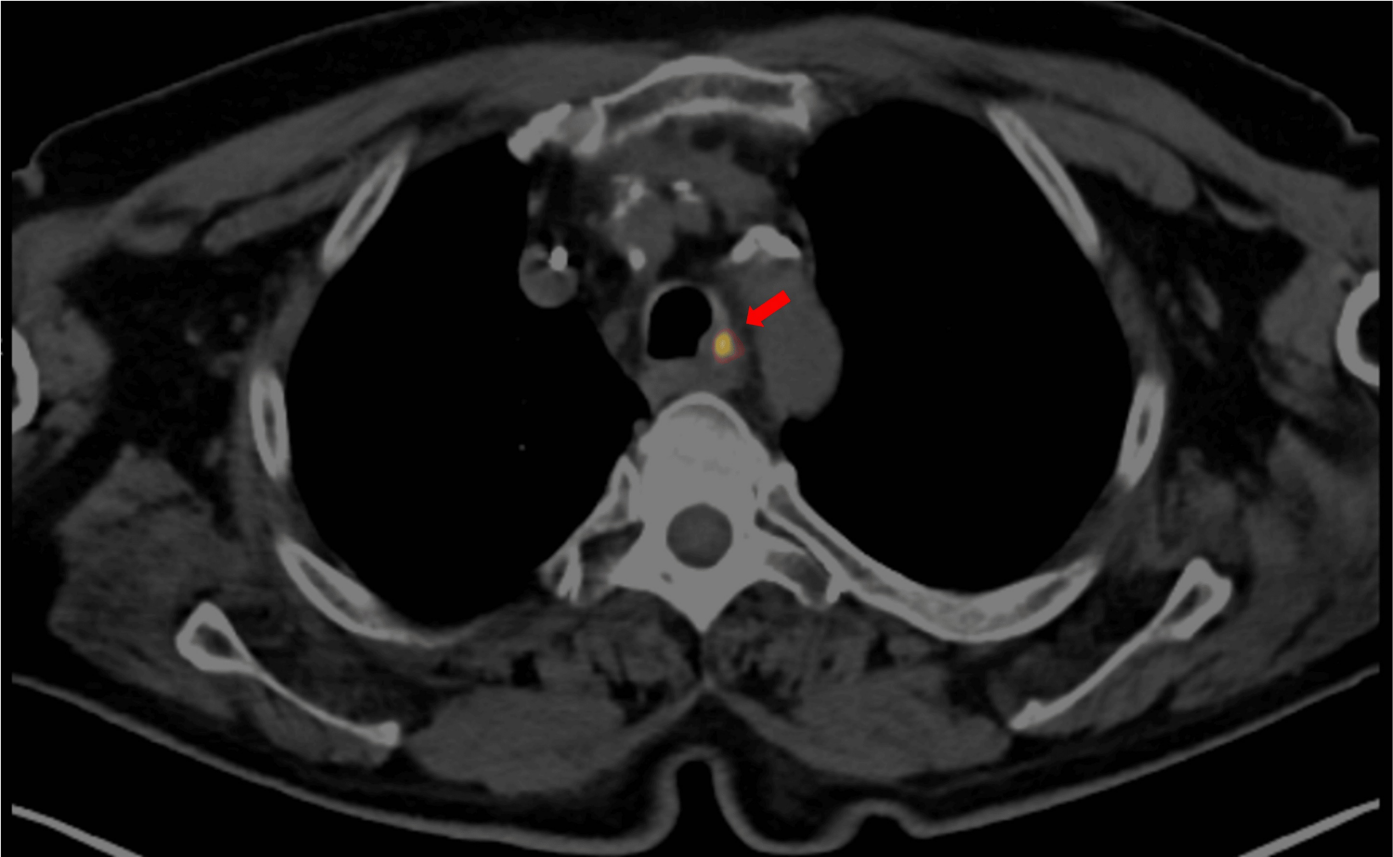
PET-CT scan with a red arrow showing a small focus of FDG uptake in the upper part of the posterior wall of the trachea, along with evidence of endotracheal irregularity PET-CT: Positron Emission Tomography-Computed Tomography; FDG: Fludeoxyglucose

A PET-CT scan conducted six months after the initial diagnosis showed resolution of the laryngeal tumor and cervical lymphadenopathy (Figure 4). However, a small focus of FDG uptake was observed in the upper part of the posterior wall of the trachea, at the site of a tracheoesophageal lymph node (Figure 5). Initially, the new focus of FDG uptake was thought to represent a tracheoesophageal lymph node. Therefore, an esophagogastroduodenoscopy (EGD) with endoscopic ultrasound (EUS) was planned. The case was subsequently discussed at the multidisciplinary (MDT) meeting, where irregularity on the tracheal mucosa raised suspicion of endotracheal metastasis, an unusual, but possible occurrence.

**Figure 6:**
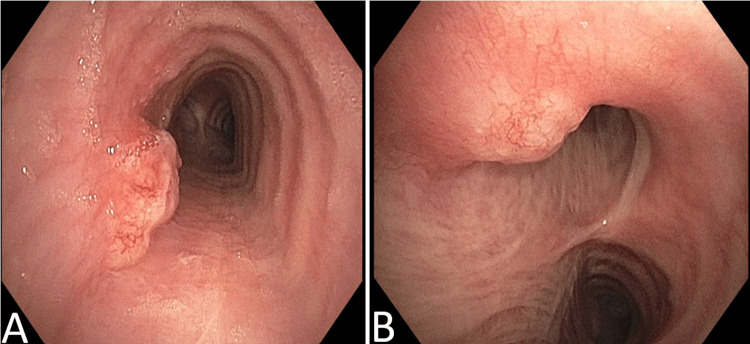
Bronchoscopic findings of two endotracheal lesions A: One lesion located in the left posterolateral aspect of the upper part of the trachea. B: Another in the left posterolateral aspect of the lower part of the trachea.

**Figure 7:**
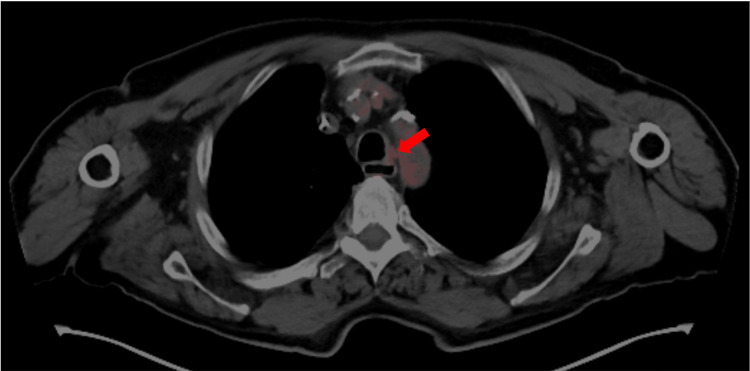
Follow-up PET-CT scan with a red arrow showing a decreased size and FDG uptake at the upper endotracheal lesion PET-CT: Positron Emission Tomography-Computed Tomography; FDG: Fludeoxyglucose

The patient underwent a bronchoscopy, which revealed two small endotracheal lesions: one in the left posterolateral aspect of the upper part and another in the left posterolateral aspect of the lower part of the trachea (Figure 6). Histopathological analysis of the biopsied specimens demonstrated invasive moderately differentiated keratinizing squamous cell carcinoma (Figure 3). Upon review at the MDT meeting, the endotracheal lesions were determined to be metastases from the primary laryngeal carcinoma, based on their similar histopathological features, temporal relationship, and the presence of multiple lesions. Treatment was initiated with a combination regimen consisting of carboplatin and fluorouracil chemotherapy along with pembrolizumab immunotherapy. A PET-CT obtained two months later, demonstrated a reduction in the size of the endotracheal lesion with decreased FDG uptake (Figure 7).

## Discussion

HNSCC encompasses a group of malignancies arising from the squamous cells lining the tissues of the head and neck region, including the oral cavity, hypopharynx, nasopharynx, oropharynx, lip, nasal cavity, paranasal sinuses, and salivary glands [1]. HNSCC ranked as the seventh most common cancer globally, with approximately 890,000 new cases annually, constituting about 4.5% of all cancer diagnoses worldwide and causing around 450,000 deaths each year [2].

Distant metastasis (DM) in HNSCC is observed in approximately 10% of cases at diagnosis, with an additional 20-30% of patients developing metastases during the disease course. The presence of DM is associated with a poor prognosis, typically reducing median survival to about 10 months. Common metastatic sites include the lungs, bones, and liver, with most occurrences arising within two years of initial diagnosis. The risk of DM increases with advanced tumor stages, extranodal extension, and specific tumor subtypes, underscoring the importance of early detection and comprehensive staging to optimize management strategies [6].

Tracheobronchial metastases, although predominantly arising from primary lung cancer, can also result from extrapulmonary malignancies, such as breast, colorectal, and renal cancers, accounting for about 2% of airway involvement cases [9]. In HNSCC, these metastases are rare and typically develop through hematogenous or lymphatic dissemination or direct extension from adjacent structures like the thyroid or esophagus. They are often classified based on their mode of development, including direct mucosal implantation, lymphatic spread to the submucosa, or hematogenous dissemination [10]. Clinically, tracheobronchial metastases may present as airway obstruction, hemoptysis, or dyspnea, with metachronous occurrences being more common than synchronous ones. It is often challenging to differentiate metastases from primary tracheobronchial malignancies, which exemplifies the significance of the disciplinary approach [11]. Moreover, tracheobronchial metastases are associated with significant morbidity and poor prognosis, highlighting the importance of vigilant surveillance in advanced HNSCC [10].

This case of HNSCC with endotracheal metastases was established through a series of investigations. A PET-CT, performed scan months after the initial diagnosis, identified a small focus of FDG uptake in the upper posterior wall of the trachea. Initially interpreted as an upper tracheoesophageal lymph node, further discussion at an MDT meeting prompted consideration of endotracheal metastasis. This led to a bronchoscopy, which confirmed the presence of two small endotracheal lesions, and biopsies of these lesions confirmed squamous cell carcinoma, consistent with metastases from the laryngeal carcinoma. The multidisciplinary approach was crucial; without it, the patient might have undergone an EGD with EUS, potentially delaying both diagnosis and treatment.

The treatment of HNSCC with tracheal metastasis requires a comprehensive, individualized approach due to the aggressive nature of the disease and its associated poor prognosis [5]. Multimodal strategies are essential, often combining systemic therapies like platinum-based chemotherapy and immune checkpoint inhibitors (pembrolizumab, nivolumab) with locoregional modalities such as stereotactic body radiotherapy (SBRT) and salvage surgery. Advances in immunotherapy, particularly in patients with PD-L1-positive tumors, have improved outcomes, as demonstrated in trials like KEYNOTE-048 [7,12]. These therapies aim to achieve disease control, enhance survival, and alleviate symptoms. The prognosis for patients with HNSCC involving endotracheal metastasis is generally poor, reflecting the advanced disease stage and aggressive tumor behavior.

## Conclusions

In conclusion, this case highlights the rare occurrence of endotracheal metastases in HNSCC, emphasizing the critical need for heightened awareness and multidisciplinary expertise for rapid recognition and management of uncommon manifestations. Despite the aggressive nature of the malignancy, multimodal treatment approaches, including concurrent chemoradiotherapy and immunotherapy, offer the potential for disease control and symptom management.

## References

[1] Barsouk A, Aluru JS, Rawla P, Saginala K, Barsouk A (2023). Epidemiology, risk factors, and prevention of head and neck squamous cell carcinoma. Med Sci (Basel).

[2] Sung H, Ferlay J, Siegel RL, Laversanne M, Soerjomataram I, Jemal A, Bray F (2021). Global Cancer Statistics 2020: GLOBOCAN estimates of incidence and mortality worldwide for 36 cancers in 185 countries. CA Cancer J Clin.

[3] Johnson DE, Burtness B, Leemans CR, Lui VW, Bauman JE, Grandis JR (2020). Head and neck squamous cell carcinoma. Nat Rev Dis Primers.

[4] Miranda-Filho A, Bray F (2020). Global patterns and trends in cancers of the lip, tongue and mouth. Oral Oncol.

[5] Pisani P, Airoldi M, Allais A (2020). Metastatic disease in head and neck oncology. Acta Otorhinolaryngol Ital.

[6] Pannunzio S, Di Bello A, Occhipinti D (2023). Multimodality treatment in recurrent/metastatic squamous cell carcinoma of head and neck: current therapy, challenges, and future perspectives. Front Oncol.

[7] Magnes T, Wagner S, Kiem D, Weiss L, Rinnerthaler G, Greil R, Melchardt T (2021). Prognostic and predictive factors in advanced head and neck squamous cell carcinoma. Int J Mol Sci.

[8] Bosetti C, Carioli G, Santucci C (2020). Global trends in oral and pharyngeal cancer incidence and mortality. Int J Cancer.

[9] Nguyen BD, Ram PC, Roarke MC (2008). Endotracheal metastasis from squamous cell cancer of the head and neck: PET/CT imaging. Clin Nucl Med.

[10] Madariaga ML, Gaissert HA (2018). Secondary tracheal tumors: a systematic review. Ann Cardiothorac Surg.

[11] Sørensen JB (2004). Endobronchial metastases from extrapulmonary solid tumors. Acta Oncol.

[12] Harrington KJ, Burtness B, Greil R (2023). Pembrolizumab with or without chemotherapy in recurrent or metastatic head and neck squamous cell carcinoma: updated results of the Phase III KEYNOTE-048 study. J Clin Oncol.

